# Unusual Right Gonadal Vein Drainage: A Journey Into Rarity

**DOI:** 10.7759/cureus.50108

**Published:** 2023-12-07

**Authors:** Aditya S Pedaprolu, Jay Dharamshi

**Affiliations:** 1 General Surgery, Jawaharlal Nehru Medical College, Datta Meghe Institute of Higher Education and Research, Wardha, IND; 2 Urosurgery, Jawaharlal Nehru Medical College, Datta Meghe Institute of Higher Education and Research, Wardha, IND

**Keywords:** renal vein, testicular vein, pelvic venous drainage, venous anomaly, inferior vena cava, ovarian vein, gonadal vein

## Abstract

The gonadal veins, responsible for draining from the paired gonads (testes in males and ovaries in females), exhibit variations in anatomy. Traditionally, the right gonadal vein directs its drainage into the inferior vena cava, while the left gonadal vein typically connects to the left renal vein. However, in the case of a 45-year-old woman diagnosed with a non-functional right kidney who underwent a right nephrectomy, an intraoperative observation revealed an unusual configuration: the right gonadal vein (ovarian) was found to drain directly into the right renal vein instead of its usual route into the inferior vena cava. This case report aims to elucidate this anomalous finding and provide a literature review on the prevalence of such anomalies in the existing research. This case report aims to raise awareness about the atypical drainage patterns of gonadal veins and underscore the importance of meticulous dissection of hilar renal vessels.

## Introduction

The venous drainage system of gonads, encompassing the ovaries in females and the testes in males, is facilitated by a pair of structures called gonadal veins. These veins manifest as the ovarian vein in women and the testicular vein (or internal spermatic vein) in men [1]. The ovarian veins originate from the pampiniform plexus, a network of veins surrounding the ovary and fallopian tube. The left ovarian vein typically joins the left renal vein, while the right ovarian vein commonly empties into the inferior vena cava (IVC) [2].

According to existing literature and available data, the embryological derivation of the right gonadal vein can be traced back to the lower segment of the right subcardinal vein. Originating from the caudal portion of the subcardinal vein, the right gonadal vein follows a course that leads to its drainage into the supra-subcardinal anastomosis. Notably, the supra-subcardinal anastomosis, along with a small segment of the subcardinal vein, contributes to the formation of the IVC. Consequently, the typical trajectory for the right gonadal vein involves direct drainage into the IVC [3]. The right renal vein is generally shorter, measuring approximately 2-4 cm, while the left renal vein is longer, ranging from 6-10 cm. Functionally, the right renal vein exclusively collects blood from the right kidney. In contrast, the left renal vein receives blood from the left kidney and accommodates veins from the left adrenal gland and gonads [4].

Various case reports have documented atypical vascular patterns originating from the kidneys, encompassing vessels branching at wide angles, bifurcating before reaching their intended destination, and encircling structures within the abdominal cavity [5]. One prevalent variation involves the duplication of gonadal veins, with a higher incidence on the left side (around 13%) than on the right side (approximately 2%). In IVC duplication, the left gonadal vein may flow into the left IVC. Additionally, gonadal veins can be partially divided, with gonadal arteries passing through them, or they may receive blood from other sources, such as a duodenal or suprarenal vein. An alternative drainage pattern may involve the gonadal veins flowing into the common iliac veins. In specific cases, the right gonadal vein has been observed to drain into the right renal vein [1].

## Case presentation

**Figure 1:**
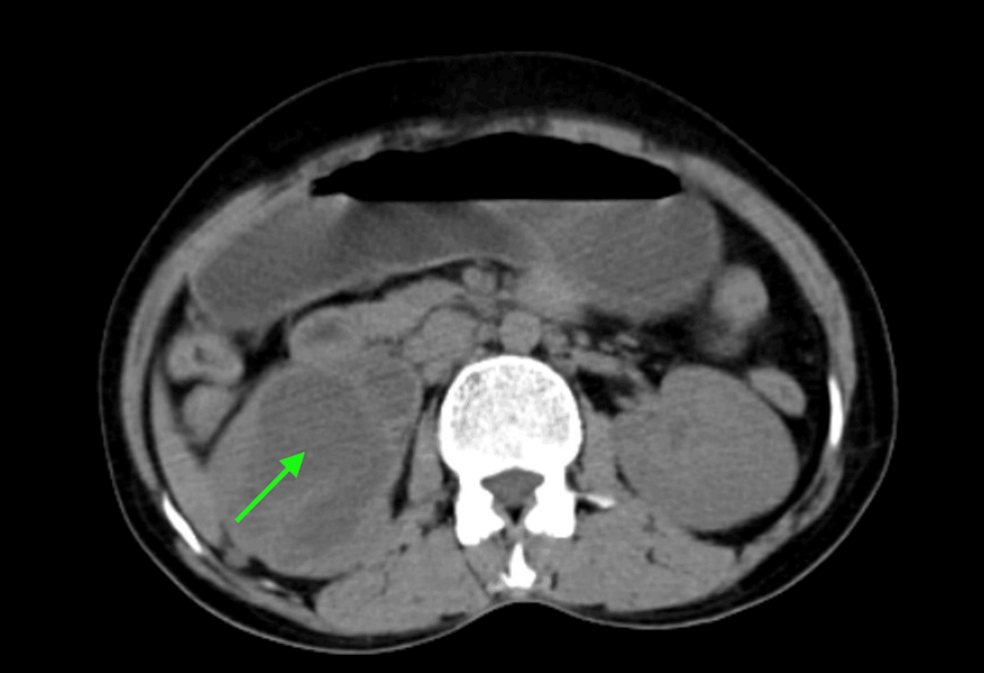
Computerized tomography of the kidney, ureter, and bladder suggesting right poorly functioning kidney with hydroureteronephrosis (green arrow).

**Figure 2:**
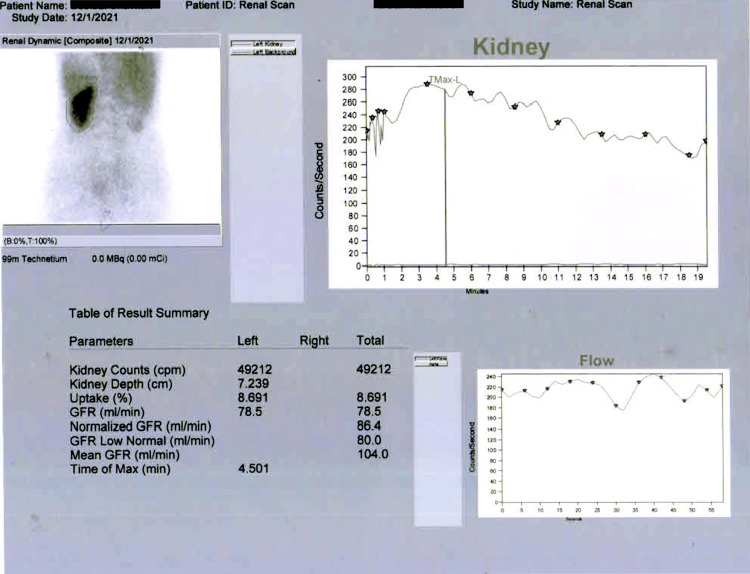
Renal scintigraphy scan showing homogeneous cortical tracer extraction over the left kidney but no significant appreciable cortical tracer extraction or tracer excretion noted throughout the study over the right kidney.

The case pertains to a 45-year-old woman presenting with symptoms that included pain in the right lumbar region of the abdomen, burning sensation during urination, frequent episodes of vomiting, and recurring fever, which had been persisting for a year. The patient underwent a clinical examination, and standard investigations were conducted after initial symptomatic management. A computerized tomography (CT) urography was subsequently performed, revealing a poorly functioning right kidney with dimensions of 10.2 x 5.8 cm. The diminished function was attributed to a mid-ureteric calculus, leading to dilation of the proximal and mid-ureter, resulting in Grade-IV right-sided hydronephrosis (Figure 1). Renal scintigraphy further indicated a non-functioning right kidney, with a total glomerular filtration rate of 78.5 mL/minute suggesting fairly preserved parenchymal function and non-obstructed drainage of the left kidney and non-functioning right kidney (Figure 2).

**Figure 3:**
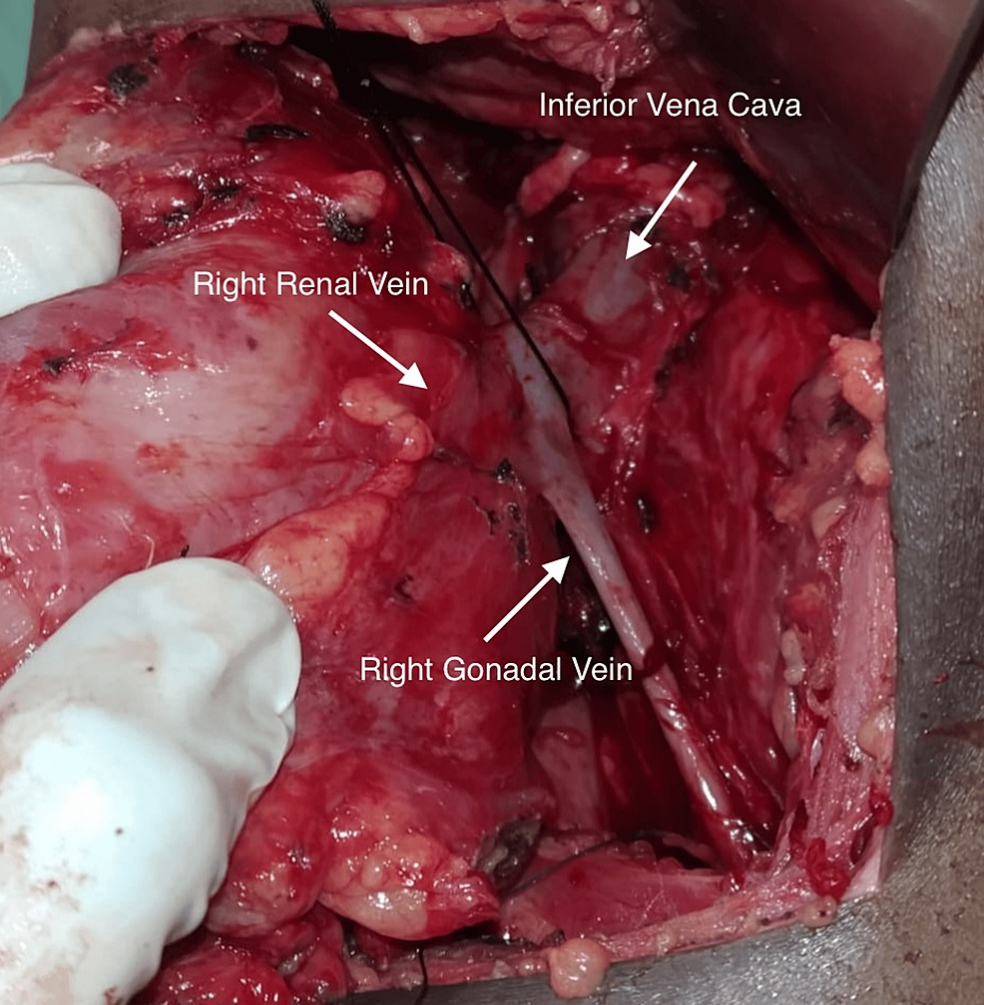
Intraoperative discovery of anomalous venous drainage of the right gonadal vein draining into the right renal vein and not the inferior vena cava.

The patient underwent a planned right-sided nephrectomy in a surgical setting that was appropriately prepared and draped under aseptic conditions. A flank incision was carefully made, progressing through the layers of the abdominal muscles. The abdominal muscles were incised, and peritoneal reflection was performed. After blunt dissection, Gerota’s fascia was identified and opened. The kidney was meticulously dissected at the hilum, and the right renal artery was dissected, separated, and ligated. An unexpected vein draining into the renal vein from its inferior aspect was observed during the dissection of the right renal vein. This vein was dissected downward, revealing that it ran alongside the right ureter and entered the pelvis (Figure 3). This finding indicated the presence of the right gonadal vein, specifically the right ovarian vein in this case.

After the identification of the right gonadal vein, the renal vein was ligated and subsequently divided. The kidney specimen was excised and forwarded for histopathological examination. Adequate hemostasis was achieved, and a lumbar abdominal drain was inserted. The incision was closed in layers, and a sterile dressing was applied. The patient underwent extubation and was transferred to the recovery room. The histopathology report indicated xanthomatous pyelonephritis. The postoperative course proceeded without complications, and the patient was discharged on the tenth day post-surgery following the complete removal of sutures. After discharge, she was placed on routine follow-up.

## Discussion

Renal vascular anomalies are generally identified either intraoperatively during any surgical procedure involving the renal system or during post-mortem. We have highlighted the unique occurrence of the right gonadal vein draining into the right renal vein in certain case studies. A retrospective study led by Koc et al. aimed to establish a potential correlation between variations in the right ovarian vein, particularly instances where it drained into the right renal vein, and the presence of pelvic varices. The study involved 324 women categorized based on the presence or absence of ovarian vein variations utilizing routine abdominal CT scans. The primary objective was to compare the occurrence and extent of pelvic varices and ovarian vein reflux between the two groups. The findings revealed that 9.9% of the women exhibited a variation of the right ovarian vein draining into the right renal vein. In comparison, 90.1% showed the typical direct connection to the IVC. Importantly, the study did not establish a significant association between variations in the right ovarian vein and the occurrence of pelvic varices [6].

In a separate case study, Krishna et al. observed 25 cadavers from the Department of Anatomy at the Sri Guru Ram Rai Institute of Medical and Health Sciences in Dehradun. Their primary objective was to document variations in renal and gonadal veins on both sides. Notably, they observed that in 8% of the cadavers, the right gonadal vein drained into the right renal vein [7]. Simultaneously, Asala et al. focused on examining testicular veins and arteries in 150 dissection room cadavers at the University of the Witwatersrand in Johannesburg and the University of Zimbabwe in Harare. In two cases, the right testicular vein drained into the right renal vein instead of the IVC [8]. These case studies indicated an incidence of approximately 8-10% of patients exhibiting this anatomical variation in the gonadal vein without any evident pathological significance related to the disease [8].

A comprehensive understanding of abdominal vessel location and anatomy is crucial for vascular surgeons, general surgeons, traumatologists, urologists, and radiologists. This comprehension plays a pivotal role in the success of surgical interventions and the precision of diagnostic procedures in radiology [9,10]. Non-invasive tools such as radiological investigations, including contrast imaging studies, can be used in preoperative planning, facilitating a thorough evaluation of anatomical structures. Still, identifying renal vascular anatomical variations can be very difficult, so thorough knowledge before proceeding with any procedure is vital [11]. 

## Conclusions

Renal vascular anomalies typically manifest without clinical symptoms, often remaining undetected until an operative procedure or autopsy reveals them. Radiologists, traumatologists, urologists, general surgeons, and vascular surgeons rely heavily on their knowledge of renal vasculature location and structure for both surgical and diagnostic purposes across various medical interventions. A comprehensive understanding of all potential congenital anomalies related to renal vasculature is imperative during surgery. In our specific case, we identified the right gonadal vein draining into the right renal vein, deviating from the typical drainage into the IVC. During meticulous dissection of renal vasculature in any surgical procedure, a thorough grasp of renal anatomy, venous drainage, and anomalies in venous drainage is essential to mitigate potential vascular complications. This study aims to illuminate abnormal anatomical variations in renal vasculature, particularly this scenario of the right gonadal vein draining into the right renal vein, as well as to highlight how surgeons can benefit from a profound understanding of these venous drainage anomalies and renal anatomy, ultimately facilitating the avoidance of complications during surgical procedures.
